# EHMTI-0080. How migraine is affected by therapies for multiple sclerosis

**DOI:** 10.1186/1129-2377-15-S1-D11

**Published:** 2014-09-18

**Authors:** C Condello, L Pinessi, L Savi

**Affiliations:** 1 Headache Center, Città della salute e della scienza, Torino, Italy

## Introduction

There are a number of available disease-modifying therapies for Multiple Sclerosis (MS). Prevalence of migraine is higher in SM patients than in the general population, so possible effects of these preventive treatments on migraine should be monitored. A review of the literature on the subject is presented.

## Methods

PubMed, Ovid and Google Scholar searches were conducted looking for Migraine combined with Beta-Interferon (bIFN), Glatiramer Acetate (GA), Natalizumab, Fingolimod, Laquinimod, Teriflunomide, Azathioprine, Methotrexate, Mitoxantrone and Ciclofosfamide.

## Results

Use of bIFN-1a was related to worsening of previously diagnosed migraine (37,5-41%, p<0.05) and to high percentage of de novo headache presentation (41-44%, p=0.05, ¼ of which with migraine features).

GA was associated with only 11% of worsening of migraine, and a direct comparison with bIFN showed the latter as much more migraine-inducing.

Effects of Natalizumab on migraine were evaluated in a small sample, but a significant reduction in migraine frequency and MIDAS scores was detected.

Only spurious cases of worsening of migraine were reported with use of Fingolimod.

## Conclusions

Of all disease-modifying therapies used in MS, bIFN is the only one which showed a clear association with worsening of previous migraine. Interpretation of data is difficult on increased incidence of migraine after starting a disease-modifying treatment, due to uneasy distinction between facilitation of a true migraine and secondary headache, attributable to medications.

Migraine should always be carefully assessed in patients affected by MS, in particular if treated with disease-modifying medications, to evaluate eventual modification of migraine itself.

No conflict of interest.

